# Back in time: Reversed speech as a window into cognitive language processing—A systematic review

**DOI:** 10.3758/s13423-026-02952-1

**Published:** 2026-08-25

**Authors:** Mathias Scharinger

**Affiliations:** Research Group Phonetics, Institute of German Linguistics, Philipps-University Marburg, Pilgrimstein 16, 35032 Marburg, Germany

**Keywords:** Reversed speech, Backward talking, Processing units, Working memory, Phonological awareness, Segmentation, Chunking

## Abstract

The speech signal unfolds in time. This has repercussions for speech production and processing. For production, processing units are assembled in a language-specific order, while in perception, the acoustic signal is segmented into specific, temporally ordered processing units. Specific insights into the forward processing of speech and its assumed mechanisms can be gained from reversed speech. Reversed speech can be either the production of speech in reverse order by skilled speakers (subjectively reversed speech), or speech played back in reverse order for investigating the mechanisms underlying speech segmentation and perception (objectively reversed speech). Direct research on reversed speech has hitherto been very sparse, leaving out opportunities to further the understanding of underlying segmentation and perception mechanisms together with temporal constraints on forward processing. To this end, a systematic literature review on reversed speech is presented here. Three insights can be gained from this review. First, findings from subjectively and objectively reversed speech can be integrated in coupled oscillator models, capitalizing on neural oscillations that align with individual speech sounds, syllable and phrases. Second, the temporal order of information in time windows < 40 ms is relatively irrelevant for perception, while the relative order of processing units is important for both perception and production. Third, objectively reversed speech is predominantly used as a control condition in speech processing studies but should be more carefully implemented, depending on the exact research questions. In general, cross-frequency coupling can be considered a domain-general neural mechanism underlying cognitive flexibility of which reversed speech is a prime candidate.

## Introduction

Spoken language is based on an acoustic signal that unfolds in time. This has repercussions for speech production and speech perception. For speech production, larger processing units such as syllables, words, and phrases have to be organized in time and coordinated according to language-specific rules (e.g., word order; Comrie, 1981). Observed word order preferences seem to be correlated with cognitive aspects of the listener (Amici et al., 2019; Hahn et al., 2020)—for example, working memory (Amici et al., 2019). On the processing level below the syllable, the speech signal is constrained by anatomical and physiological factors: For most speech sounds in the languages of the world, a regulated pulmonal air volume stream is required, which depends on controlled exhalation (Lieberman & Blumstein, 1988; Reetz & Jongman, 2008), causing the vibration of the vocal folds in the larynx and the generation of a subsequent filtered larynx signal by means of articulation (Reetz & Jongman, 2008). On this level, the physiological processes are very hard to reverse in time. Recent research has demonstrated that aside from a fine-tuned coordination of muscle activity (Jürgens, 2002), motor timing processes are essential for speech production (Kotz & Schwartze, 2015; Tremblay et al., 2015).

Aside from spoken language, temporal ordering and reordering of processing items is essential for optimizing goal-directed behavior (Hartmann et al., 2022). The ordering of items in working memory is constrained by working memory capacity (Miller, 1956), a constraint that is counterbalanced by interactions between prefrontal control regions and sensory cortex that allow dynamic reconfigurations of task goals (Buschman, 2021; Buschman & Miller, 2007). A proposed neural mechanism for these network-level interactions are cortical oscillations and the cross-coupling between them (Aktürk et al., 2022; Riddle et al., 2020, 2021). Reordering of processing items can thus be considered a part of cognitive flexibility subserving goal-directed and adaptive behavior, including spoken language communication.

For speech perception, several models assume a segmentation mechanism to portion the acoustic signal in optimally sized processing units (Mattys & Bortfeld, 2016). The temporal extent of these units is cognitively constrained in that certain memory systems (e.g., sensory memory or working memory) have transient properties: Information withheld there decays after specific time intervals (Baddeley, 1992, 2000; Schröger, 2007; Winkler et al., 1993; Zlotnik & Vansintjan, 2019). Neurobiological models assume at least two time windows into which the acoustic signal is segmented and analyzed (“tracked”): a larger window (125–333 ms; corresponding to syllables) and a smaller window (20–40 ms; corresponding to speech sounds; Poeppel, 2003). Notably, the assumed neural mechanism associated with the syllable-analysis window (auditory theta-oscillation at 3–8 Hz; Ghitza & Greenberg, 2009) has found to be coupled with a motor-related mechanism (motor theta-oscillation at 3–8 Hz; Assaneo & Poeppel, 2018). This adds to the evidence that motor processes or general-purpose timing mechanisms play an important role in speech perception (Devlin & Aydelott, 2009; Kotz & Schwartze, 2015). Note that speech processing is a prominent case for studies on cross-frequency coupling. This is because the coupling of cortical frequencies is functionally related to multiple-item and sequence encoding, synchronization for long-distance communication (e.g., between frontal and sensory cortical regions) and the temporal parsing of continuous stimuli (Giraud & Poeppel, 2012; Hyafil et al., 2015).

For both speech production and speech perception, reversed speech might offer a unique window into processing details and the nature of the underlying mechanisms. Reversed speech here refers to two phenomena, related to either production or perception, and can be termed “subjectively reversed speech” and “objectively reversed speech.”

### Subjectively reversed speech

Subjectively reversed speech is the outcome of a specific skill by persons who can fluently read and speak language from right-to-left in left-to-right systems such as English or German. Persons with this skill are referred to as *backward talkers* (Cowan et al., 1982). Backward talkers would easily produce the word “laughter” as “rethgual” (if their strategy is letter-based) or “retfal” (if their strategy is speech-sound-based).

Backward talking is not in the center of linguistic or neurocognitive research. The phenomenon is usually considered in relation to language games in childhood (Conklin, 1959) or the development of secret languages, following a relatively easy principle of reversing segments for arriving at incomprehensible language (Chrisman, 1893). In linguistic research, it has been found that the reversal of segments and sounds in a principled and consistent way is limited to short sequences (Moreton et al., 2021). As discussed by Moreton et al. (2021), complete word-reversals or phrase-reversals are unknown word-formation operations in the languages of the world. Attested, by contrast, are repetitions of segments or segment sequences, most prominently seen in reduplications (e.g., “bye-bye”).

### Objectively reversed speech

**Fig. 1:**
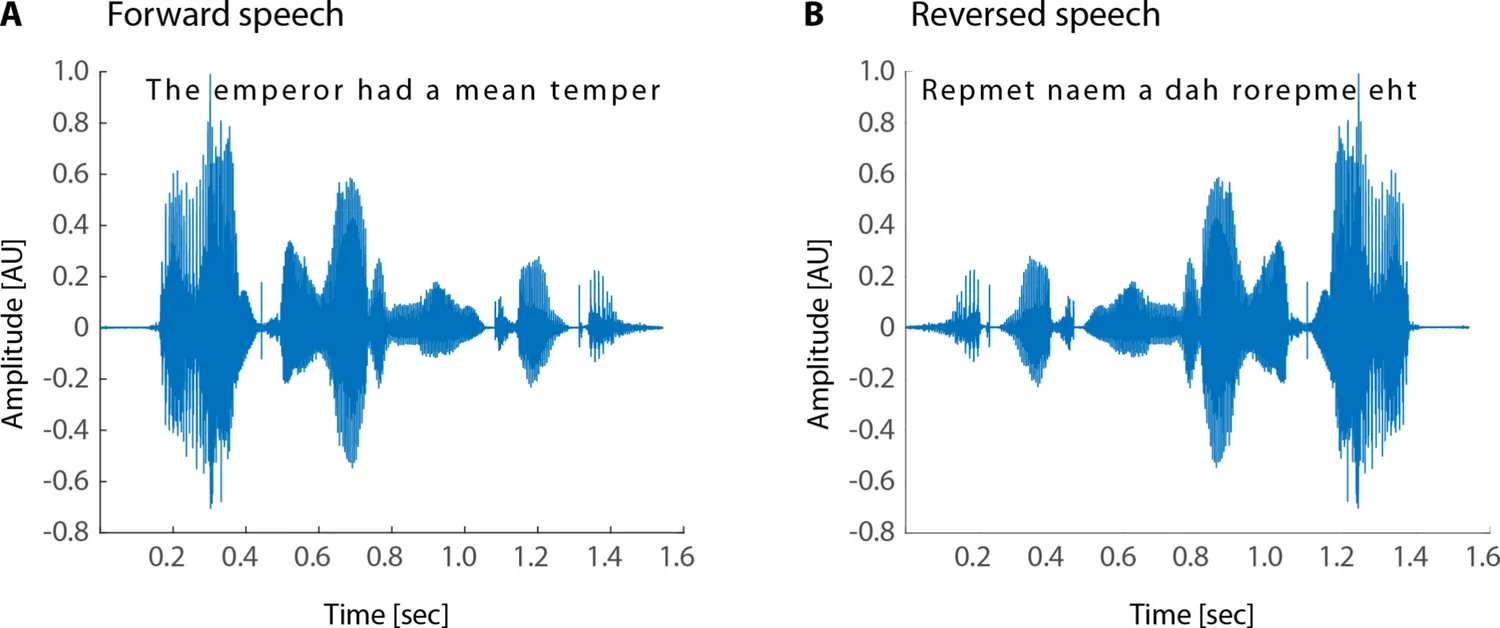
Illustration of forward (**A**) and reversed (**B**) speech. The forward speech example stems from the TIMIT database (e.g., https://osf.io/z4cmy/files/es9dv). The panels shows the wave forms—that is, amplitude samples per time unit (bound between the arbitrary units [AU] − 1 and + 1). The reversed speech is the result of a reordering (reversal) of the amplitude samples per time unit, using the function flip in MATLAB (e.g., https://osf.io/z4cmy/files/hge47)

Objectively reversed speech is the outcome of specific speech manipulations. On a global level, the reversal can affect an entire recording or sound file, which is either played backwards or in which the order of amplitude samples per time unit is reversed (flipped). Reversed speech by means of a sample-by-sample reversal is illustrated in Fig. 1, based on a speech recording from the TIMIT corpus (Garofolo et al., 1993). In this example, the original sentence “The emperor had a mean temper” was reversed using the *flip* function of MATLAB (MATLAB R2024a, The MathWorks, Inc, Natick, MA; code available on https://osf.io/z4cmy/).

**Fig. 2:**
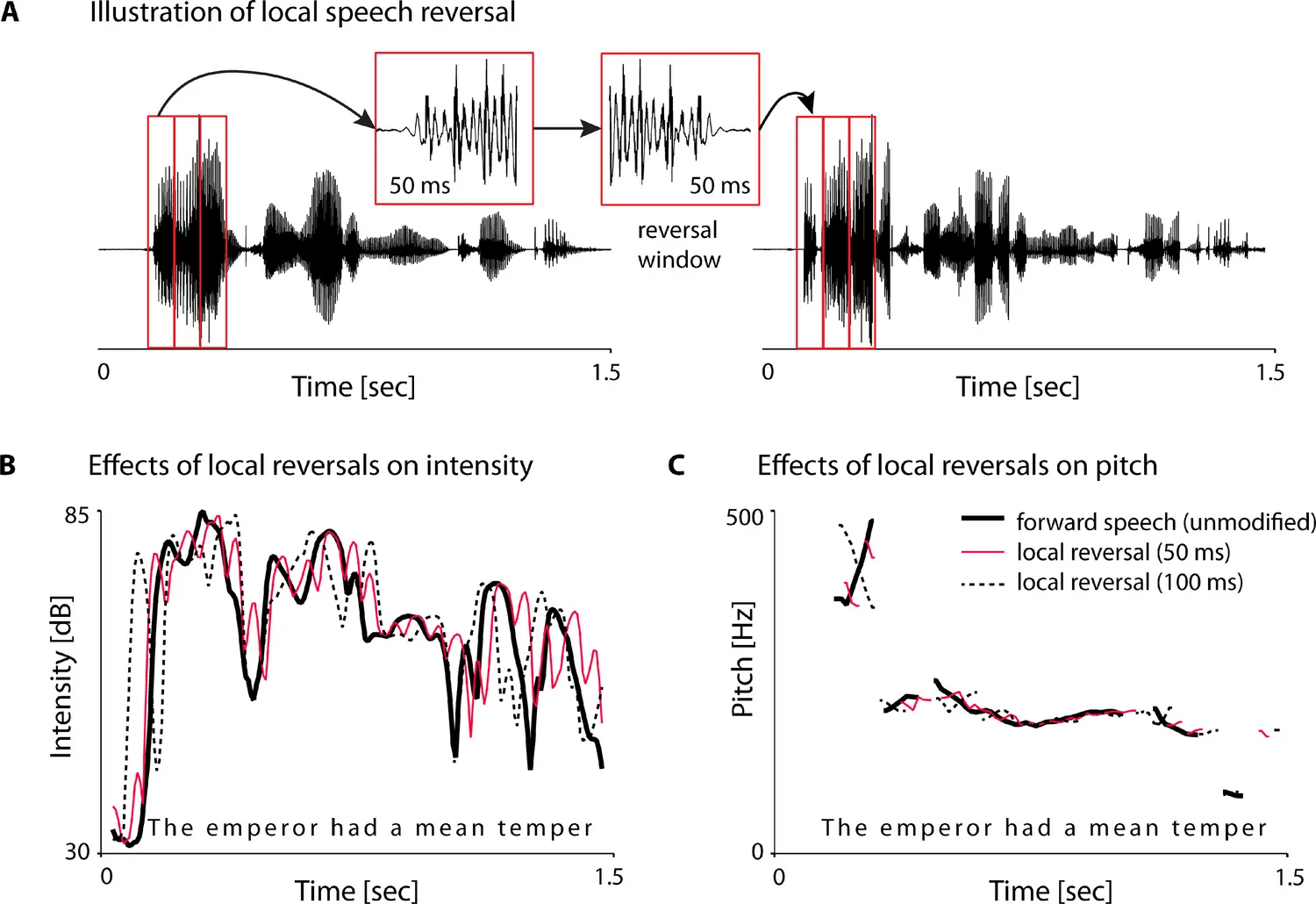
Illustration of locally reversed speech. **A** The original (forward) speech signal is divided into equally sized windows (here, 50 ms). The amplitude samples in these windows is reversed. The windows with the reversed signal are concatenated in the same order as taken from the original speech signal. The resulting signal consists of consecutive windows in which the speech signal is reversed (e.g., https://osf.io/z4cmy/files/5byvr). **B** Illustration of the effects of local reversals on the intensity (corresponding to the amplitude envelop) of the speech signal. With increasing reversal window sizes, the intensity (amplitude) modulation gets disrupted (e.g., for reversal in 100-ms windows, https://osf.io/z4cmy/files/t5emp). **C** Illustration of changes in the pitch contour as a function of increasing reversal window size. Similar to intensity, the pitch modulation gets disrupted with increasing reversal windows, but altogether, pitch is more robust against local reversals because pitch modulations have a lower rate than amplitude modulations in speech

On a local level, the reversal can be applied to consecutive time windows. In these time-windows, the order of amplitude samples is reversed, but the global order of the time windows’ sequence is retained. This is illustrated in Fig. 2.

Objectively reversed speech is often used as a control condition in speech perception or auditory perception experiments. As global (word-based or sentence-based) reversals, local reversals maintain long-term spectral properties of the original speech signal, but with increasing reversal sizes, they affect amplitude and pitch modulation (i.e., the amplitude envelop and the fine structure of the running spectrum are distorted).

With regard to perception, it has been found that local reversals in windows up to 50 ms are still intelligible (Saberi & Perrott, 1999). Local reversals based on larger windows (≥ 100 ms) result in less intelligible speech (Saberi & Perrott, 1999).

### This study

Subjectively and objectively reversed speech has rarely been investigated in its own right. Regarding subjectively reversed speech, there are some studies on individuals with excellent skills in talking backwards (Cowan et al., 1985), but only few studies on languages other than English. Objectively reversed speech has either been used as control material in auditory and speech perception experiments, or as test material for investigating constraints on speech perception mechanisms. Research on these two types of reversed speech hardly ever attempted to bridge the realms (e.g., by looking at individuals who show excellent backward talking skills and testing their abilities to understand objectively reversed speech). To structure a possible future field of investigations, this study is based on a systematic literature review on reversed speech. The hope is that a global view on reversed speech (including subjective and objective reversal) furthers insights into speech processing mechanisms that may be shared in production and perception. Furthermore, the study attempts to couch the speech-specific aspects in a domain-general perspective of cognitive processing.

## Materials and methods

The current work consists of a systematic literature review following the PRISMA guidelines (Page et al., 2021). The literature search was conducted using the Web of Science (WoS; https://www.webofscience.com), an online platform providing access to several databases of reference and citation data across the disciplines of natural and social sciences as well as arts and humanities. Six WoS collections (covering around 170 million records) were selected: Web of Science Core Collection, Current Contents Connect, KCI Korean Journal Database, Medline, Research Commons, SiELO Citation Index, accessed on February 10, 2026). The search was based on the complex search string shown in (1):

(1)“backward speech” or “reverse* speech” or

“backward talk*” or “reverse* talk*” or “backward speak*” or “reverse* speak*” or “backward pronunciation” or “reverse pronunciation” or “backward articulat*” or “reverse articulat*” (TOPIC).

**Fig. 3:**
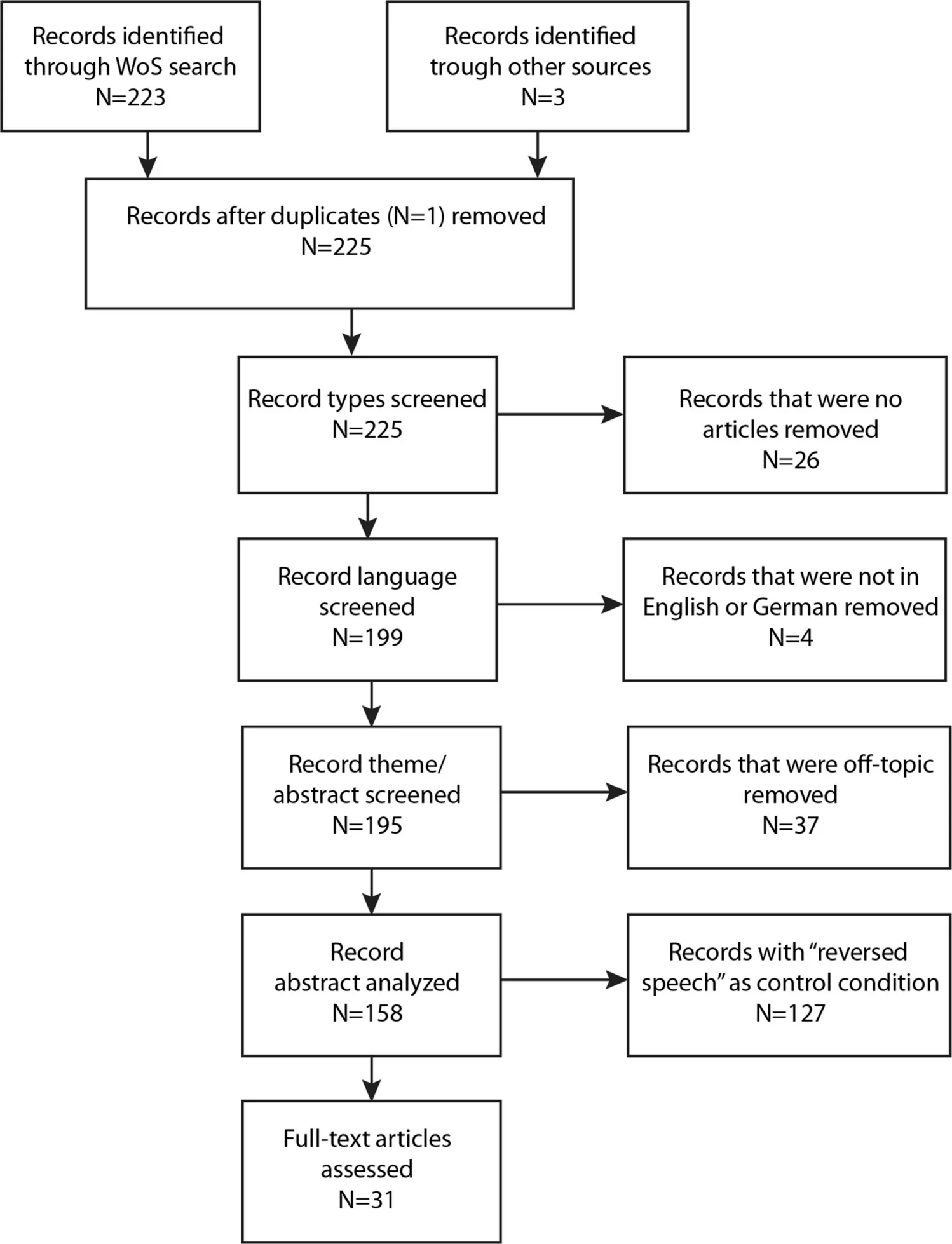
Illustration of processing steps of the systematic literature analysis, following the PRISMA-guidelines

The search term resulted in 223 records which were exported in the BibTex-format and analyzed in JabRef (https://www.jabref.org/) and BibTex. Three additional records were added obtained through a google scholar search (https://scholar.google.com/). The entire set of records was then screened for duplicates (*N* = 1), records not categorized as»article« (*N* = 26), records in languages other than German or English (*N* = 4). The resulting set of records (*N* = 195) was thematically analyzed on the basis of their abstracts. Records not focusing on speech or language were removed (*N* = 37). The resulting 158 records were further analyzed thematically and divided into two broad categories: (a) references relating to studies in which reversed speech serves as a control condition (*N* = 127) and (b) references relating to studies in which reversed speech is of primary interest and in the focus of the research (*N* = 31). Full-text articles of the latter set of records underwent a closer qualitative analysis. The processing steps according to the PRISMA-guide lines are illustrated in Fig. 3.

## Results

### Objectively reversed speech as a control condition

A total of 127 records referred to studies in which objectively reversed speech (on a global level) was used as control condition. Global reversals refer to the flipping of amplitude samples of an entire recording (see Fig. 1).

Objectively reversed speech on a global level, as illustrated above, is usually unintelligible, but maintains several properties of forward speech, such as spectral details and intensity (Brown et al., 2012), but also voice-related properties, such as mean fundamental frequency and voice-quality measures such as jitter and shimmer (Sheffert et al., 2002). However, one has to bear in mind that with short stimuli being reversed, intelligibility might be less affected than desired. As discussed below, reversals in short time windows (< 50 ms) can be still intelligible. Additionally, reversals affect different speech sounds differently, as discussed in Ishida et al. (2025a) in relation to local reversals. On a global level of reversal, vowels are still comprehensible due to their steady-state nature, which is maintained, albeit reversed in time. As Brown et al. (2012) conclude, reversed speech might not be the best control condition, at least not for studies where differential activation between speech and nonspeech conditions are of interest.

The analysis of records pertaining to studies in which reversed speech is used as a control condition helped identifying categories regarding methods and population foci (see Table 1). Most of the identified studies are psychoacoustic studies on adults, children and patients.

**Table 1:** Overview of studies in which reversed speech is used as control condition

Method	Focus	Studies	Total
Psychoacoustics	Adults	27	**39**
	Patients	8	
	Children	4	
Behavioral recognition task	Adults	14	**28**
	Patients	6	
	Children	8	
functional Magnetic Resonance Imaging (fMRI)	Adults	9	**26**
	Patients	10	
	Children	7	
Electroencephalography (EEG)	Adults	17	**22**
	Patients	3	
	Children	2	
Positron Emission Tomography (PET)	Adults	5	**5**
functional Near-Infrared Spectroscopy (fNIRS)	Children	4	**4**
Electrocorticography (ECoG)	Adults	3	**3**

In the psychoacoustic studies, objectively reversed speech is either used as masker or distractor. A representative study in this realm is the work by Summers and Molis (2004) who analyzed the effect of competing speech and speech-like maskers on recognition, comparing normal-hearing with hearing-impaired participants. They demonstrate that informational masking is most effective if it is based on comprehensible (i.e., nonreversed) speech.

The next category subsumes studies based on behavioral experiments focusing on speech or speaker perception, therefore, involving higher processing levels with influences from lexical, syntactic and speaker-related information. One representative study (Jones et al., 2012) analyses the impact of irrelevant (distracting) speech on lexical retrieval. The authors showed that task-irrelevant speech is effective only if it provides access to meaning (i.e., if it is not reversed). Tsantani et al. (2016) and Perrachione et al. (2019) use reversed speech as control condition for voice processing experiments. Tsantani et al. (2016) provide evidence that low-pitched voices were preferred over higher-pitched voices, irrespective of whether participants were tested with forward or reversed speech. Further, the authors showed that the ratings of trustworthiness of a particular voice did not depend on forward or reversed speech. Perrachione et al. (2019) showed that dissimilarity ratings for different voices did not depend on forward versus reversed speech. An important insight from these studies is that reversed speech is relatively unintelligible, but still carries enough information for speaker voice recognition.

In studies based on functional magnetic resonance imaging (fMRI), language and speech functional areas of the human brain are identified. Researchers capitalize on the blood-oxygen-level dependent (BOLD) signal, correlating with the metabolic energy supply by oxygenated blood in respective brain areas. The BOLD response is usually used in contrast design, where a speech related condition is compared to a resting state condition, or to a condition in which speech-like, nonintelligible stimuli are presented. One such stimulus type is reversed speech. A representative study in this realm is the work by Binder et al. (2000). The authors compared the cortical activation patterns in response to noise, frequency-modulated tones, reversed speech, pseudowords and words. Forward speech, reversed speech and pseudowords elicited stronger activations in bilateral superior temporal sulcus (STS) compared to frequency-modulated tones. This finding is interpreted as evidence that the STS is engaged by speech and speech-like stimuli, irrespective of whether the stimuli are meaningful or intelligible.

However, using reversed speech as control or comparison condition is not undisputed, as briefly alluded to before. Apart from Brown et al. (2012), Stoppelman et al. (2013) compared different baselines in this regard and observed that reversed speech was not the best condition in order to dissociate speech-specific from basic auditory responses. This is because in their study, the comparison between speech and reversed speech eliminated many activation centers in frontal and temporal areas. This did not hold for signal-correlated (modulated) noise. When used as baseline in comparison with speech, primary auditory responses could be removed from the response pattern. Furthermore, the authors could show that reversed speech led to a faster decline of activation in frontal areas of the brain, most likely reflecting top-down attenuation in cortical processing once the signal is classified as unintelligible.

Another category of records subsumes studies using electroencephalography. In these studies, reversed speech is also used as control condition, for instance, in experiments capitalizing on the synchronization of electric brain activity with the speech signal (“entrainment”). Deng and Srinivasan (2010) tried to test the model proposed by Poeppel (2003) who proposes two distinct time-scales of speech processing, correlating with distinct oscillation frequencies, 40 Hz for phonemes, and 4–16 Hz for syllables. Deng and Srinivasan found that the brain’s oscillation frequencies between 4–16 Hz from left-hemispheric regions anterior to primary auditory cortex were correlated with the amplitude envelop of the speech signal. This correlation held only when speech was presented forward, not when it was presented backwards (i.e., reversed). This suggests that cortical tracking of speech requires “forward” transitions between speech sounds and consecutive syllables, which are compromised when reversed.

Finally, reversed speech was also used in several brain imaging studies based on positron emission tomography (PET), functional near-infrared spectroscopy (fNIRS) and electrocorticography (ECog). The function of reversed speech in these latter types of studies is very similar to the exemplary studies using fMRI briefly discussed above. Regarding PET, Wong et al. (2002) found that reversed speech, compared to a silent baseline, engaged prefrontal regions in the human brain. In a fNIRS study, targeting very young children’s cortical processing, Sato et al. (2012) found that maternal (forward) speech elicited stronger activation than maternal reversed or nonnative speech in left temporo-parietal regions, while nonnative forward speech did not differ from nonnative reversed speech. Reversed speech as control condition in fNIRS studies is however disputed (Mushtaq et al., 2019). Similarly, among the few studies using the ECog method, there is substantial critique on reversed speech as control condition (Brown et al., 2012). In the same vein as Stoppelman et al. (2013), it is argued that a better control for teasing apart basic auditory from linguistic processing is signal-correlated noise (Brown et al., 2014).

In sum, the reviewed studies show that objectively reversed speech is predominantly used as a control condition in order to learn more about forward speech. Reversed speech is, to certain degrees, useful for distinguishing meaningful speech from speech-like stimuli without meaning. In this regard, reversed speech behaves similar to pseudowords or to nonnative speech. If, however, the focus is on differentiating basic auditory from linguistic (i.e., language-specific spoken word or sentence) processing, reversed speech is less useful. Depending on the specific contrasts used in these studies, reversed speech as compared to forward speech may show overlapping activity in either auditory (temporal) regions or linguistic (fronto-temporal or fronto-parietal) regions.

### Reversed speech as research focus

Articles in which reversed speech is of primary interest can be categorized into the two categories elucidated in the introduction: subjectively and objectively reversed speech. The 31 articles analyzed in more detail in this review have been published between 1903 and 2025.

### Subjectively reversed speech

The majority of articles on subjectively reversed speech focus on a rare skill of some people who can produce fluent backward speech. Cowan and Leavitt (1982) describe the skill of talking backward of two boys (ages 8 and 9) and establish the onset of this particular ability by focusing on case reports of 27 adult backward talkers. The emerging generalization is that the ability starts between age 7 and 12, coinciding with orthography acquisition and increasing phonological awareness. The authors distinguish between two bases for backward talking: orthography and phonology. Backward speech based on orthography would start out from reversed letters in words or sentences, pronounced according to the language-specific grapheme-to-phoneme conversion rules; for instance, the sequence < ou > in »house« would surface as [uo]. Backward speech based on phonology would initiate from reversed speech sounds (phonemes) in words or sentences; in this case, the sequence < ou >, denoting the phoneme [au], would remain [au] in backward speech. The authors report that backward speech is usually based on reversing units (graphemes or phonemes) in words with a maximum number of eight segments, and only in rarer cases, units are reversed in whole sentences. That is, backward speech means that word order in sentences is maintained, while segment order in words is reversed. Cowan and Leavitt (1982) demonstrate that among the two strategies (orthographic and phonemic), the phonemic strategy led to slightly better performance of backward talking. It is concluded that the ability of talking backwards, particularly if based on phonemes, requires (increased) phonological awareness and an explicit knowledge of letter-to-sound correspondences. Additional working-memory and verbal-skill tests administered by Cowan and Leavitt (1982) suggest that the children’s skill covaried with verbal skill rather than with working memory capacity. Cowan and colleagues also characterize the backward talking skills of an adult (Cowan et al., 1982), whose backward speech was phoneme-based, but occasionally showed “orthographic contaminations” (Cowan et al., 1982, p. 51). The latter term was used by the authors to describe deviations from a phoneme-based reversal (which would maintain the phoneme’s auditory quality); for instance, < had > would become [da], not [dæ^h^]. Notably, the fluent reversal of segments (up to 10) was word based, while word positions in sentences were not altered. The memory skills of the backward talker only slightly exceeded average scores. Notably, backward-talking skills improved upon practice. This was illustrated in revisiting the competence of the backward-talking boys some years later (Cowan & Leavitt, 1987).

In a later article, Cowan and colleagues integrated the existing knowledge on backward talking and provided a theoretical account (Cowan et al., 1985). Therein, they analyzed 10 backward talkers using an orthographic strategy and 10 backward talkers using a phonemic strategy. As in earlier studies, the authors report orthographic influences in those using a phonemic strategy for backward talking and arrived at a model wherein a “metaphonological” system as additional representation layer is assumed. This system is based on phonology but individually influenced by orthography, and serves as source for representations used in backward speech. Notably, representations from the metaphonological system are units that can be sequenced in working memory. The skill of talking backward is thus assumed to be based on the nature of representations in the metaphonological system (correlating with phonological awareness), and on working memory.

A first multimethod study on backward talking was carried out by Prekovic et al. (2016) who investigated members of a Serbian family qualifying as fluent backward talkers without explicit training. The authors assumed the skill to be a trait and combined cognitive, neurophysiological (EEG), brain imaging (fMRI), and genetic analyses. Focusing on one family member, cognitive tests suggested good working memory skills and electrophysiological data implied that segment reversals in words preceded semantic integration. The brain imaging data revealed three regions particularly subserving back speech processing, the fusiform gyrus, left prefrontal gyrus, and right motor area. A genetic analysis on six family members suggested that the neural processes involving the aforementioned regions may be mediated by a mutation in the RIC3, RIPK1, and ZBED5 genes. The molecular and biological bases of the RIC3 in relation to the skill of backward talking were described in a later study in more detail (Pradhan et al., 2024). Altogether, Prekovic et al. (2016) conclude that the skill of talking backward may indeed be an individual trait, capitalizing on excellent working memory skills, supported by frontal cortex and left fusiform gyrus.

In a similar vein, Torres-Prioris et al. (2020) conducted a multimethod study on two Spanish backward talkers. The authors stressed the possible advantage for backward talking stemming from languages with a shallow orthography (i.e., where the grapheme–phoneme relation is direct, as is the case for Spanish). As in previous studies, backward talking was not solely dependent on memory skills but also on phonological skills. In particular, Torres-Prioris et al. (2020) could demonstrate that the backward talkers had increased grey matter in fusiform gyrus, as well as in middle and inferior frontal gyrus (corroborating and extending the findings by Prekovic and colleagues). Furthermore, the backward talkers showed differences in the dorsal fiber tracts; in particular, the connectivity between insular cortex and fusiform gyrus was enhanced. Altogether, the findings suggest structural differences (enhanced grey matter and connectivity) in the phonological cortical network of the expert backward talkers, corroborating earlier research which highlighted the importance of phonological sequencing skills underlying the specific skill of (fluently) talking backwards. Due to the essential involvement of the fusiform gyrus, previous findings that illustrated the modulatory effect of orthography could also be accommodated. The fusiform gyrus as high-level visual processing area has previously been identified as being relevant for visual word processing (Tsapkini & Rapp, 2010), but is also supposed to support phonological and more abstract orthographic processing (Zhao et al., 2017). In this regard, the area is a prime candidate for supporting processes in the metaphonological system established by Cowan and colleagues.

Some studies report the particular skill of talking backwards to co-occur with mental illness. One of the first reports is the observation from the neurologist Silas Weir Mitchell (1903) who described talking backward as abnormal speech characteristics of a patient with brain tumor (postmortem analyzed as tumor in the left anterior lobe). A later study focused on a patient with a head injury over left frontal areas who started to read, write and speak backwards (Jokel & Conn, 1999). The author attempted to classify this as symptom of conversion disorder and suggested several causes, based on motor-related impairments, impairments in spatial cognition or vision. In a latter study, backward speech was classified as symptom of a clinical condition without a necessary organic basis (Jokel & Wolf, 2017).

A typological account is given by Moreton et al. (2021). The authors asked whether subjectively reversed speech is used as potential linguistic rearrangement processes. Linguistic rearrangements are predominantly seen in reduplications (i.e., the repetitions of syllables, parts, or combinations thereof; e.g., “bye-bye” or “helter-skelter”). Linguistic rearrangements based on reversals, however, are not attested in languages of the world. Moreton et al. (2021) hypothesized that reversals might be learnable, but would be too taxing for working memory during online processing, in contrast to repetitions. Therefore, reversals are disfavored as means of linguistic rearrangement. Moreton et al. (2021) provided experimental evidence for effortful, nonautomatic processing of reversal as compared with repetition (reduplication) patterns. They also argued that an additional difficulty of reversal patterns would be that phonotactic sound co-occurrence constraints were violated (e.g., yielding the consonant cluster [dl] from the original word < bald >).

#### Objectively reversed speech

Despite the observation that reversed speech is usually unintelligible (and therefore used as control condition in studies on speech processing), several studies have provided evidence that local reversals of speech do not compromise intelligibility. In the study by Saberi and Perrott (1999), participants reported perfect intelligibility at reversal windows of 50 ms, while at 100 ms, intelligibility dropped to 50%. The authors interpret their findings as providing evidence to the view that the modulation envelops between 3 and 8 Hz are essential for speech intelligibility. Note that modulation frequencies in speech relate to the dynamic changes of amplitude and/or frequency values. The aforementioned modulation between 3 and 8 Hz concerns the amplitude envelop of speech, correlating with syllable rate (Ding et al., 2017; Varnet et al., 2017). Syllable nuclei usually consist of vowels which show the highest acoustic intensity in the speech signal and thus essentially contribute to the amplitude envelop. Modulation frequencies between 3 and 8 Hz are relatively stable cross-linguistically (Ding et al., 2017), although peak modulations and modulation bandwidths may differ between languages, most likely due to different syllable structures (Varnet et al., 2017). In modulation frequencies between 3 and 8 Hz, signal parts with durations of 1/8 (125 ms) to 1/3 (333 ms) are amplified (Greenberg et al., 2003; Stilp et al., 2010) and correspond to syllable durations. It is assumed that amplitude modulation frequencies of speech lead to synchronization of brain oscillations in the same frequency band (3–8 Hz, theta-band) (Ghitza, 2012, 2013; Poeppel, 2003). The theta oscillation is considered to reflect a cortical segmentation and chunking mechanism. Information in temporal integration windows corresponding to the oscillation duration (the inverse of the respective modulation frequency) is discretized and thereby optimized for further processing. It is thus reasonable to assume that reversal windows in locally reversed speech bear a relation to (amplitude) modulation frequencies. Stilp et al. (2010) suggest that this relation exists, in that effects of local reversals and filtering low-frequency modulation frequencies shows comparable effects on intelligibility (Experiment 1 of Stilp et al., 2010). They conclude that peaks in modulation frequencies roughly correspond to one-half of the periodicity construed by local reversals. A 6.25-Hz modulation frequency would therefore correspond to an 80-ms reversal window.

Subsequent studies disputed the relation between modulation frequencies and reversal windows. Ishida et al. (2025a) demonstrated that a direct translation of the effects introduced by local reversals in fixed time windows into modulation frequencies corresponding to these windows is not possible. The patterns of intelligibility decreases with increasing reversal windows and filtering of decreasing modulation frequency cut-offs are not comparable and are differently moderated by language-based syllable structure, syllable rate, and speech sound type (Ishida et al., 2025b; Stilp et al., 2010). Ishida et al. (2025b) showed that Japanese words are more tolerant to temporal distortions introduced by local reversal than English words. The reason, the authors argue, lies in the different syllable structure of the two languages: English, as a stress-timed language, has very complex syllables, with consonant clusters in syllable onsets and codas, while Japanese, as a mora-based language, has rather “simple” syllables, following a regular consonant–vowel (CV) pattern. Local reversals in English may therefore affect more segments in a syllable than in Japanese and thereby reduce intelligibility to a higher degree. By contrast, Ueda et al. (2017) observed no intelligibility differences between English, German, Japanese, and Mandarin Chinese for window sizes shorter than 20–40 ms, particularly when the segment durations were normalized with respect to the average in each language. This suggests two things: First, the effect of syllable rate (Stilp et al., 2010) on comprehending objectively reversed speech can be accommodated by time-normalization, and second, there seems to exist a relatively stable window between 20 and 40 ms wherein the flipping of acoustic information has no detrimental effect on perception.

Several authors report a lexical advantage, paralleling an language familiarity benefit for locally reversed speech. Intelligibility of locally reversed speech for reversal window sizes close to 40 ms was better for native (familiar) than second language (Kiss et al., 2008) and better for words than for pseudowords (Ishida, 2021). Ishida (2021) and Ishida et al. (2025b) demonstrated that local reversal affect different speech sounds to different degrees. Local reversals particularly affected speech sounds that were either brief (fricatives such as [s]) or temporally asymmetric (plosives such as [t]), due to, for example, characteristic formant transitions, which are compromised when reversed. Given that fricatives and plosives usually have temporal extents between 40 and 70 ms (Abramson & Whalen, 2017; Jongman, 1989), reversal windows corresponding to their average duration and covering transitions to neighboring speech sounds are arguably more detrimental than the same reversal windows are to longer speech sounds, such as vowels. Vowels, by contrast, are characterized by relatively long steady-state phases, the reversal of which does not compromise spectral cues.

The most important argument against a direct comparison of reversal window sizes and modulation frequencies concerns the different outcome of the manipulation: Local reversals result in an unnatural flipping of acoustic information, detached from articulatory plausibility, while filtering of modulation frequencies compromises landmarks or cues to forward-periodicities in the speech signal. Nevertheless, there has been attempts to combine effects of local reversals and modulation filtering by chimeric stimuli. Matsuo et al. (2020) split band-passed filtered sentences into a high and a low-frequency band, using six cut-off frequencies. Local reversals in three different time windows were either applied to the lower or the higher frequency band, resulting in chimeric stimuli. They showed that intelligibility decreased with increasing reversal windows in the chimeric stimuli and a widening of the spectral range over which the reversals were applied. Comparing the effects of local reversals in chimeric and nonchimeric stimuli, they demonstrated that intelligibility was best when the amplitude envelop in the frequency range from 840 to 1600 Hz was preserved. A similar result was found by Ueda and Matsuo (2021). Note that another comparison of modulations is given in the study by Nakajima et al. (2018). They used local temporal reversals at several window sizes (20–320 ms) alongside spectral distortions (“mosaic speech”) similar to noise-band vocoding. Their results corroborated the findings that reversals up to 40 ms are perfectly intelligible. Additionally, they could show that, based on the same segment duration, temporal distortions affected intelligibility more than spectral distortions.

It has been argued that the cortical theta frequency, correlated to syllable rate, may provide the upper limit of the reversal windows sizes beyond which locally reversed speech becomes unintelligible (Ghitza & Greenberg, 2009). This view has been challenged, for example, by Remez et al. (2013) and Grataloup et al. (2009), who point out that the critical reversal window size between 40 and 70 ms above which restoration of locally reversed speech crucially deteriorates is much smaller than the typical syllable duration (200 ms) in languages of the world (Ding et al., 2017). Remez et al. (2013) explored human modulation sensitivity by comparing local reversal based on natural and on sine-wave speech and concluded that “sensitivity to modulation, independent of timbre, simply develops far more quickly than the syllable, arguably at the pace of the phonetic segment” (Remez et al., 2013, p. 1356). Ueda and Ciocca (2021) resolve the apparent disparity in integrating their findings on locally reversed speech into the framework proposed by Poeppel and colleagues (Chait et al., 2015; Giraud & Poeppel, 2012; Luo & Poeppel, 2007; Poeppel, 2003; Poeppel et al., 2008). According to this framework, speech perception is based on at least two time scales, one corresponding to the syllable (125–333 ms), the other one corresponding to the phoneme (20–40 ms). Correspondingly, the tracking and integration of the syllable-sized and phoneme-sized information is proposed to be supported by cortical oscillations with oscillation period times corresponding to unit durations—that is, covering the frequencies from 3 to 8 Hz (theta) for syllables and from 25 to 50 Hz (beta and lower gamma). Ueda and Ciocca (2021) argue that the extraction of envelop information in syllabic windows is contingent on the integrity of information in the phonemic window. Integrity is affected considerably when local reversal are based on window sizes exceeding the phonemic window size.

The aforementioned framework can also accommodate the findings by Kohlmann (1984), who showed that rhythm perception of reversed speech is similarly based on syllabic units than natural (forward) speech. Participants had to reproduce the perceived speech rhythm of natural and reversed sentences in German, English, French and Japanese, using a morse key. The number of detected syllabic events did not differ between forward and reversed speech, suggesting that retrieval of syllabic structure information was not comprised, despite the unintelligible signal. By contrast, reversed speech does not effectively prime the meaning of words (Kreiner et al., 2003) or unconsciously affect listeners (Begg et al., 1993). As evidenced in many studies using reversed speech as control condition, this type of speech is usually completely unintelligible.

Objectively reversed speech on a global level does not seem to compromise nonlinguistic information. Several studies have shown that voice identity discrimination and recognition abilities are not affected when the speech materials for each voice is presented in reverse (Ming et al., 2024; Schweinberger, 2001; Sheffert et al., 2002). Ming et al. (2024) analyzed voice identity discrimination in sighted and blind people, using forward and reversed speech in native and a nonnative language. Voice identity discrimination was possible for both forward and reversed speech, for sighted and blind people. While discrimination deteriorated for reversed compared with forward speech, no such differences were found when nonnative speech was used. Sheffert et al. (2002) demonstrated that familiar and unfamiliar talkers can be recognized from reversed speech. Participants were trained on speech or reversed speech and tested on reversed speech or natural speech. Talker identification was always possible, but accuracy was reduced when reversed speech was used as training material. Schweinberger (2001) used voice priming in a famous versus unfamiliar voice decision task. Voice primes, presented in reversed speech, were as effective primes than voice primes presented in natural (forward) speech.

Finally, three articles provide rather general accounts on objectively reversed speech. Kellogg (1939) describes his impression of listening to speech played backwards. The overall impression is that reversed speech sounds “foreign” and is obviously not intelligible. In his attempt to transcribe reversed speech, he discusses difficulties in the English grapheme-to-phoneme correspondences and position-dependent pronunciations that are affected in backward speech (e.g., “silent” [h] at word beginnings or omitted [t] at word endings). He closes with the observation that information longer than individual speech sounds might be better learnable in their reversed version and that backward music might still allow to recover the original melody. Meyer‐Eppler (1950) provides a more phonetically inspired, short account of reversed speech where he differentiates between small effects of reversed vowels and large effects of transient sounds such as plosives. He illustrates reversed speech by a phonetic transcription with examples from German and concludes that reversed speech may be learnable as any other foreign language.

## Discussion

The systematic review on reversed speech has shown that the topic can be approached from at least three different perspectives: 1) subjectively reversed speech, 2) objectively reversed speech, and 3) reversed speech as control condition. These three perspectives are summarized in the following paragraphs. The discussions concludes with a synthesis, including a model sketch, and outstanding questions for future research.

### Subjectively reversed speech

Subjectively reversed speech refers to the (exceptional) ability of individuals to fluently talk “backwards”—that is, to reverse sounds or letters in words or sentences. With 11 records, the literature on subjectively reversed speech is relatively scarce. From the existing research, one can conclude that the ability of talking backwards is gradual, ranging from the mere reversing of letters in words (e.g., “words” resulting in “sdrow”) to the phonetic reversal of articulations on the sentence level (e.g., “The cat is on the mat” resulting in “(h)tam eth no si tac eth”; “h” denoting the aspiration usually following the consonant “t”). There is a discernible training effect, particularly for the phonetic reversals. The onset of the ability is the beginning of literacy (i.e., when children learn to read and to write), demonstrating a strong orthographic influence of backward talking (Cowan et al., 1985). From the literature review provided here it becomes evident that one of the best predictors of fluent backward talking is phonological awareness (Cowan et al., 1985). Defined as the degree of sensitivity to the sound structure of oral language (Anthony & Francis, 2005), phonological awareness is also a crucial predictor of developing reading and writing skills (i.e., for the acquisition of orthographic competence; Castles & Coltheart, 2004; Gillon, 2004). Increased phonological awareness in preschoolers covaries with their subsequent success in learning to read and write. Vice versa, once orthographic knowledge has been acquired, phonological awareness can be enhanced further (Mann, 1986). The additional boost of phonological awareness at the onset of the acquisition of orthographic knowledge seems to favor the emergence of backward talking abilities (Cowan & Leavitt, 1982, 1987; Cowan et al., 1985). The emerging picture is that backward talking is based on increased phonological awareness and the improved skill of labeling chunks of the continuous speech signal with orthographic codes (i.e., letters). Some researchers attempt to relate phonological awareness to the fine-tuning of the brain’s oscillatory dynamics (Goswami, 2018). Goswami (2018) suggests that successful speech encoding as a crucial basis for phonological awareness relies on the parsing of continuous speech into phonological processing units, supported by brain oscillations tracking syllables and stressed positions within words. Backward talkers offer an interesting perspective on such accounts because they emphasize that the successful encoding of speech does not only involve the chunking of the speech signal into processing units but crucially profits from the ability to assign labels to these processing units. This ability seems to emerge prior to the acquisition of orthography, but is strengthened by acquiring knowledge about the labels (i.e., letters). Thereby, phoneme–grapheme correspondences can be strengthened, ultimately helping to establish stable phonological and meta-phonological representations (Cowan et al., 1985), supported by auditory cortical regions and the fusiform gyrus (Dietz et al., 2005). The multimethod accounts by Prekovic et al. (2016) and Torres-Prioris et al. (2020) corroborate the latter findings.

### Objectively reversed speech

Objectively reversed speech refers to the flipping of recorded speech on either a global level (i.e., concerning entire recordings) or a local level (i.e., concerning consecutive fixed time windows), the order of which is maintained but the speech signal inside the windows is flipped. The literature is dominated by studies on locally reversed speech (15 out of 20 papers in this category; see Table 2). The most cited paper is the seminal study of Saberi and Perrott (1999; see Table 3).

**Table 2:** Overview of articles for which the research focus is on reversed speech

Category	Subcategory	Population	Articles
Subjectively reversed speech	Backward talking	Healthy	7
		Impaired	3
	Typology		1
Objectively reversed speech	Local reversals	Healthy	15
	Global reversals	Healthy	5

**Table 3:** The five most cited papers from the systematic literature research presented here

Author/Year	Area	Total citations	TC per year
Saberi and Perrott (1999)	Objectively and locally reversed speech	159	5.68
Sheffert et al. (2002)	Objectively and globally reversed speech	86	3.44
Schweinberger (2001)	Objectively and globally reversed speech	37	1.42
Cowan et al. (1985)	Subjectively reversed speech	30	0.71
Cowan et al. (1982)	Subjectively reversed speech	20	0.44

Counts according to Web of Science (https://clarivate.com/academia-government/scientific-and-academic-research/research-discovery-and-referencing/web-of-science/)

The observation that information in the temporal range between 20 and 40 ms seems robust against temporal reversals may be due to the *recoding* of information (in the sense of Miller, 1956) when transitioning from sensory to working memory. Miller (1956) suggested that the bottleneck of continuous information processing can be overcome by recoding, whereby temporal continuous information is “chunked up” into (phoneme-sized) processing units which subsequently can be combined into larger informational structures. Importantly, the relevance of Miller’s proposal lies in the fact that working memory is relatively insensitive to the amount of information per chunk (Baddeley, 1994). This accommodates the observation that at this stage of processing, it does not matter whether the information within this chunk adheres to its expected temporal direction (i.e., it may well be reversed). What fluent backward talkers also support is Miller’s often-cited working memory constraint of about seven or slightly more items that can be maintained there. The review provided here demonstrates that backward talking is mostly based on a number of chunks (phonemes) close to the aforementioned “magical” number. In the same vein, the observation that backward talking (i.e., rearranging chunks of spoken language in real time) profits from good working memory capacities is in line with Miller’s proposal and subsequent research (Baddeley, 1992).

Differences in intelligibility decrease with lengthening reversal windows may be affected by participants’ language (Ishida, 2021; Ishida et al., 2018, 2025a, 2025b) or speaking rate (Stilp et al., 2010). The latter influence can be compensated if the time axis of the reversal window is normalized (Ueda et al., 2017), the former influence seems to be based on different proportions of consonants and vowels in the syllable of different languages, with English allowing for consonant clusters in syllable onsets and codas while Japanese avoids these clusters and adheres to a consonant–vowel structure with mora-timing (Ishida et al., 2025a, 2025b). Consonants and vowels are differently affected by reversals in general. Vowels, with acoustically and articulatory steady-state phases of 90–200 ms (Ladefoged, 1996), are relatively intelligible if reversed, while plosive consonants, such as [t] in “time,” show a stark decline of intelligibility if reversed. Plosive consonants are acoustically and articulatory asymmetric, since a closure phase is followed by a release burst, aspiration and formant transition phase. The reversal of these sounds compromises the temporal cues and results in an acoustic signal which would go back to unlikely or impossible sequence of articulation gestures. Fricatives, such as [s] in “sing,” occupy a middle position in this regard. The friction noise of these sounds is relatively unaffected by reversals, as long as the reversal windows do not affect the relative temporal sequence of the fricatives’ context.

Based on the suggested relation between reversal window sizes and modulation frequencies in speech (Ishida et al., 2025a; Stilp et al., 2010), several authors have interpreted the ability to understand locally reversed speech with window sizes < 40 ms on the background of cortical oscillatory mechanisms. There is good evidence that speech processing capitalizes on temporally varying excitability of neuronal assemblies (Henry et al., 2014; Zoefel & VanRullen, 2015), giving rise to functional interpretations of the brain’s oscillation patterns (Chait et al., 2015; Giraud & Poeppel, 2012; Luo & Poeppel, 2007; Poeppel, 2003; Poeppel et al., 2008). The work of Poeppel and colleagues suggests that the oscillation patterns covary with the temporal extent of processing units, in that the cortical theta-frequency (3–8 Hz) supports syllable tracking (with average syllable durations between 125 and 333 ms), and that the beta/low-gamma frequency (25–50 Hz) aligns with phoneme processing (with average phoneme durations between 20 and 40 ms). The systematic review on reversed speech adds to the evidence of the functional importance of these two frequency bands. For speech to be intelligible, it is important that acoustic information in the longer (syllabic) time window is not time-reversed. This might be due to the fact that theta from auditory cortical regions (auditory theta) is coupled with theta from motor regions (motor theta) at around 4.5 Hz (Assaneo & Poeppel, 2018). One might consider auditory theta to be asymmetric regarding the temporal dimension of its cycles, and similarly, motor theta to be asymmetric regarding syllable production. The latter asymmetry seems to be rooted in the production mechanism itself, being based on controlled exhalation, a process that cannot be (entirely) reversed due to anatomical and physiological constraints (Lieberman & Blumstein, 1988; Reetz & Jongman, 2008). For this reason, backward talking on the basis of completely reversing articulation is not possible. Therefore, cortical oscillations may provide a mechanism which links processing in production and perception.

An oscillatory account can also accommodate findings on globally reversed speech. As remarked by Kellogg (1939), reversed speech still sounds like speech, albeit rather like a foreign language, for which reason the recruitment of (parts) of the speech processing network is not surprising. In particular, reversed vowels can still be recognized, in turn allowing speaker recognition (Sheffert et al., 2002). Speaker recognition relies on information integrated in longer temporal windows, in particular, prosodic information such as stress patterns and intonation contours. This integration seems to be supported by slow oscillations < 1 Hz (Frühholz et al., 2020). When information in windows >1 s is reversed (i.e., windows corresponding to entire phrases or sentences), voice-related and speaker-related information can still be integrated into the processing, while the temporal order of syllabic and segmental events is compromised, mismatching with the expectation of forward articulatory gestures. The entire regime of cortical oscillations is thought to be (hierarchically) connected by cross-frequency coupling (Giraud & Poeppel, 2012; Hyafil et al., 2015). Oscillations on a fast frequency (i.e., gamma or beta, 15–80 Hz) provide the cortical readout of local excitation patterns. Slower frequencies (i.e., theta or delta, 1–8 Hz) may provide the mechanism by which distant brain regions (e.g., frontal and parietal) interact and communicate with each other and by which discretized processing units (e.g., syllables) are temporally sequenced. Cross-frequency coupling appears to be not a speech-specific mechanism but seems to apply in a domain-general way in order to optimize goal-directed behavior by re-arranging and prioritizing items in working memory, depending on task demands (Aktürk et al., 2022; Riddle et al., 2020, 2021).

### Reversed speech as control condition

Among the identified records in this systematic literature review, more than 80% relate to studies in which (objectively) reversed speech is used a control condition. Reversed speech is speech-like, but not (entirely) intelligible. For this reason, its use as control condition allows to tease apart aspects of processing related to acoustics from those related to sound–meaning mappings. Most studies which used reversed speech as control condition either focused on psychoacoustic aspects (e.g., by examining to what degree reversed speech can mask forward speech), or are based on brain imaging where activation patterns were analyzed in a contrastive approach, comparing activations elicited by forward speech to those elicited by backward speech. This approach can “regress out” the contribution of more basic auditory processing from speech perception.

Due to the differential effects of reversals on different speech sounds (with consonants being more affected than vowels), the use of reversed speech as control condition is disputed. Some studies have provided evidence that reversed speech processing does not only (or exclusively) reflect basic auditory processing stages. Depending on the reversal window size of objectively reversed speech, intelligibility is also affected by participants’ native language. Therefore, in accordance with Brown et al. (2012) and others, (objectively) reversed speech as control condition must be carefully evaluated against possible confounding factors identified above.

### Conclusion and future directions

Reversed speech can be used as a window into speech processing. The systematic literature review on this topic has identified some key issues, but also outstanding future questions. Subjectively and objectively reversed speech are two sides towards looking at speech processing. Subjectively reversed speech as ability to talk “backwards” is based on the reversing of processing units (either speech sounds or letters). Usually, the information within these units is not reversed. This contrasts with objectively reversed speech, which is based on global or local reversals and where reversals in windows < 50 ms are still intelligible. Coupled oscillator models of speech processing (Ghitza, 2011; Giraud & Poeppel, 2012) with cross-frequency coupling (Giraud & Poeppel, 2012; Hyafil et al., 2015) may accommodate both aspects of reversed speech. The basic assumptions of such a model are summarized in Table 4.

**Table 4:** Overview of a proposed coupled oscillator model for integrating findings from reversed speech

Time window	Oscillation	Function	Subjectively reversed speech	Objectively reversed speech
20–40 ms	Gamma (25–60 Hz)	Integrating acoustic–articulatory information; cortical readout of excitation patterns	Reordering limited to speech sounds or letters	Intelligibility maintained when information reversed
125–333 ms	Theta (3–8 Hz)	Sequencing & coordinating syllables; ordering, reordering and prioritizing items in working memory	Flexible coupling to gamma for more flexible planning	Coupling differences based on rhythmic class of the language
500–2,000 ms	Delta (0.5–2 Hz)	Integrating prosodic & speaker-related information; synchronization of information across larger temporal scales (0.5–2 s) and across more distant brain regions (frontal-parietal)	Prosodic contours and speaker-related information are not altered	Prosody and speaker-related information are not compromised

The aforementioned models propose several functional oscillation frequencies, the period duration of which corresponds to the average duration of linguistic units such as speech sounds, syllables, or phrases. Within each period of these frequencies, information is discretized: In perception, the continuous speech signal is segmented and integrated in respective time windows, while in production, the time windows are “planning” units for temporal coordination (Goldstein et al., 2009). Being competent in reversed speech seems to rely on a more flexible coupling or de-coupling of these processing frequencies. In production of reversed speech, accessing speech sound units is likely to be detached from their syllable or phrase-based order (decoupling of gamma [25–50 Hz] and theta [3–8 Hz] or delta [0.5–3 Hz]), or a decoupling of motor and auditory theta (Assaneo & Poeppel, 2018; Rimmele et al., 2021), possibly subserved by larger working memory capacities or stronger speech unit representations. In perception, the gamma frequency (25–50 Hz) provides a possible fixed integration window wherein the temporal order of acoustic information is irrelevant. Differences in intelligibility of locally reversed speech seems then to be based on differences in the coupling of gamma and syllable-based theta frequencies. Differences in coupling most likely go back to differences in rhythmic structure of languages (e.g., mora-, syllable or stress-timed). Finally, low-frequency oscillations (delta, 0.5–2 Hz) support the integration of information in larger time-windows, such as prosody (intonation contours) and speaker-specific idiosyncrasies (Frühholz et al., 2020; Sammler et al., 2015). Reversals in these time-windows (500 ms– 2 s) are possible in production if they are based on smaller units (speech sounds). In perception, by contrast, reversals cause speech sound information to be compromised, while prosodic and speaker-related information is retained. Cross-frequency coupling in this model sketch is the functional integration of information of one level of the processing hierarchy into the next higher level. For instance, assuming gamma to provide the readout of cortical excitation patters (or spectro-temporal receptive fields), theta integrates these patterns into the syllables and accounts for their relative temporal ordering. Subsequently, delta integrates information from syllables into metrical structure and phrases.

Future research should attempt to focus on the following outstanding questions:

For instance, do skills in backward-talking directly correlate with skills in perceiving objectively reversed speech? Does the ability of backward-talking co-vary with rhythmic aspects of the native language of backward talkers (mora, syllable, or stress-timed languages)? To what degree can the prosody (intonation contours) be reversed in backward talking? Can the stronger coupling or decoupling of gamma and theta frequencies, as proposed here, be really observed during perception of objectively reversed speech or the production of subjectively reversed speech? Is the reversal window of 20–40 ms really language-independent and can the language-based differences be explained by differences in gamma–theta coupling? Assuming that cross-frequency coupling is a domain-general cognitive mechanism, does competence in backward talking and understanding objectively reversed speech covary with cognitive flexibility?

The focusing on these and related questions will surely prove fruitful for a better understanding of speech processing in a unified framework, integrating aspects of production and perception. Reversed speech is a useful window to illuminate some key aspects of this integration.

## Data Availability

Bibliographic data, search protocols, exemplary audio files and scripts are available without restrictions (https://osf.io/z4cmy/).

## Declarations

### Open practices statement

The data of all experiments, including bibliographic data, search protocols, exemplary audio files and scripts are available without restrictions (https://osf.io/z4cmy/).
